# A recessive genetic screen for host factors required for retroviral infection in a library of insertionally mutated *Blm*-deficient embryonic stem cells

**DOI:** 10.1186/gb-2007-8-4-r48

**Published:** 2007-04-03

**Authors:** Wei Wang, Allan Bradley

**Affiliations:** 1 Department of Cell Biology and Genetics, College of Life Sciences, Peking University, Beijing 100871, PR China; 2 The Wellcome Trust Sanger Institute, Hinxton, Cambridge CB10 1SA, UK

## Abstract

**Background:**

Host factors required for retroviral infection are potential targets for the modulation of diseases caused by retroviruses. During the retroviral life cycle, numerous cellular factors interact with the virus and play an essential role in infection. Cultured embryonic stem (ES) cells are susceptible to retroviral infection, therefore providing access to all of the genes required for this process to take place. In order to identify the host factors involved in retroviral infection, we designed and implemented a scheme for identifying ES cells that are resistant to retroviral infection and subsequent cloning of the mutated gene.

**Results:**

A library of mutant ES cells was established by genome-wide insertional mutagenesis in *Blm*-deficient ES cells, and a screen was performed by superinfection of the library at high multiplicity with a recombinant retrovirus carrying a positive and negative selection cassette. Stringent negative selection was then used to exclude the infected ES cells. We successfully recovered five independent clones of ES cells that are resistant to retroviral infection. Analysis of the mutations in these clones revealed four different homozygous and one compound heterozygous mutation in the mCat-1 locus, which confirms that mCat-1 is the ecotropic murine leukemia virus receptor in ES cells.

**Conclusion:**

We have demonstrated the feasibility and reliability of this recessive genetic approach to identifying critical genes required for retroviral infection in ES cells; the approach provides a unique opportunity to recover other cellular factors required for retroviral infection. The resulting insertionally mutated *Blm*-deficient ES cell library might also provide access to essential host cell components that are required for infection and replication for other types of virus.

## Abstract 2

A recessive genetic screen of an insertionally mutated Blm-/- ES cell library identifies host factors required for retroviral infection, and confirms that mCat-1 is the ecotropic murine leukaemia virus receptor in ES cells.

## Background

One characteristic of all viruses is dependency for replication on components synthesized by host cells. All types of virus are able to subvert the machinery in the host cell for replication of the viral genome and expression of viral gene products [1]. Retroviral replication has a unique aspect, namely conversion of genomic viral RNA into cellular DNA, which has been exploited for the development of antiretroviral drugs. Integration of a retrovirus into the host genome concludes the early stage of the life cycle, after which the virus can begin to multiply [2].

Study of the host factors that are involved in the retroviral life cycle is important if we are to gain a detailed understanding of the interaction between virus and host cell components. Essential host cell components are potential targets for antiviral therapies that could be developed. Many viruses have small genomes, and so the repertoire of components that can be exploited as pharmaceutical targets is very limited. Moreover, because of their rapid replication, variants in the viral genome that overcome the effect of inhibitors will be rapidly selected, diminishing the effectiveness of antiviral agents. The main drugs currently used to treat HIV infection are inhibitors of two viral proteins, namely the reverse transcriptase and the protease (encoded by the viral *pol *and *gag *genes, respectively). Also, inhibitors of the HIV-1 entry and fusion steps have been used as a third drug class in recent years [3]. Thus, therapeutic molecules targeting retroviral host factors would be a potential new route to modulation of diseases caused by retroviruses. Evidence of the importance of host factors is provided by individuals who harbor homozygous mutations in the gene encoding CC chemokine receptor (CCR)5, who are extremely resistant to HIV infection. As a result of these observations, human antibodies to CCR5 and small-molecule CCR5 antagonists are being investigated as potential HIV therapies [1,4].

Retroviral vectors are widely used as genetic vehicles or as mutagens in embryonic stem (ES) cells. Comparatively few studies have described the molecular components that are essential for the interaction between retroviruses and ES cells. In previous studies, several host genes required for viral infection were identified by screening a gene trap library constructed in somatic cells [5]. Here we describe a genetic screen designed to identify host factors in ES cells that are required for the early phase of the retroviral life cycle. This recessive screen was conducted in a library of insertionally mutated *Blm*-deficient ES cells. The random insertional mutations in this library were generated using a recombinant retroviral gene-trap vector, integration into genes of which predominantly produces a loss of function mutation; the integrated proviral DNA provides a sequence tag for identifying the mutation [6]. In principle the genome-wide gene-trap mutations in this library should provide access to mutations in the subset of genes expressed in ES cells [7]. The *Blm *(which encodes Bloom's syndrome protein)-deficient genetic background of these ES cells is the second important feature of this mutation library. Recessive genetic screens in a diploid mammalian genome require an approach to generate cells with homozygous mutations, which increases the complexity of most genetic screens because of the low rate of loss of heterozygosity (LOH) of single allelic mutations in wild-type ES cells. However, *Blm*-deficient ES cells have a 20-fold increase in the rate of LOH [8], which offers a major advantage in recessive screens. Indeed, two reports have described successful use of *Blm*-deficient ES cells to identify recessive mutations in genes required for DNA mismatch repair [9] and the glycosylphosphatidylinasitol-anchor biosynthesis pathway [10].

For the screen described here, we have confirmed the utility of this system in generating genome-wide homozygous mutations to facilitate recessive genetic screens *in vitro *by identifying *mCat-1 *as a critical gene in ES cells that is required for retroviral infection. This screen was conducted by superinfection with a retroviral vector carrying the *puro-Δtk *(puromycin-Δ-thymidine kinase) gene, a positive/negative selectable marker [11]. Clones surviving negative selection were shown to be resistant to retroviral infection, and in every case the *mCat-1 *gene was mutated. This success demonstrates the feasibility of conducting genome-wide negative selection screens for genes that confer resistance to infection.

## Results

### Screening strategy for infection resistant mutants

**Figure 1:**
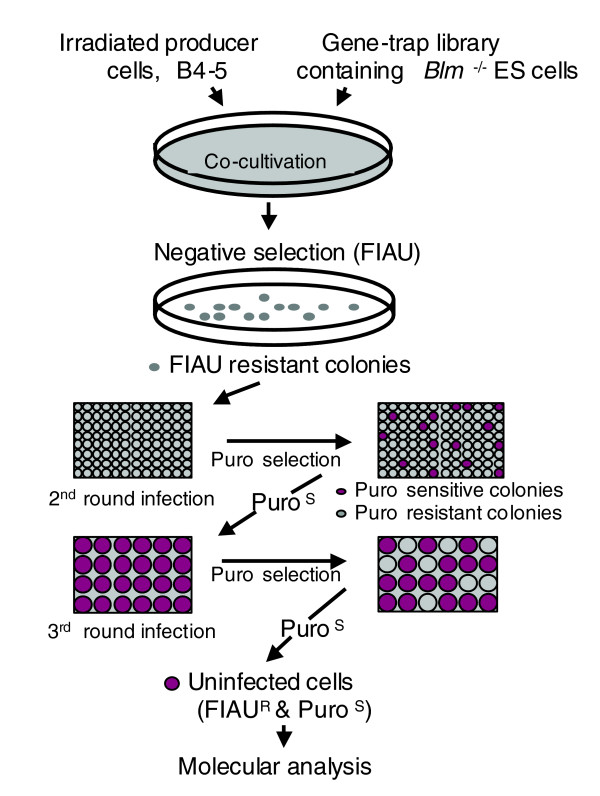
Negative selection screen for infection-resistant cells. A gene-trap library in *Blm*-deficient embryonic stem (ES) cells was co-cultivated with the irradiated viral producer cell line B4-5 and selected in 1-(-2-deoxy-2-fluoro-1-β-D-arabino-furanosyl)-5-iodouracil (FIAU). Single cell clones surviving the primary negative selection screen were tested twice by exposure to viral supernatant and selection in puromycin to confirm whether they were resistant to infection. The confirmed infection-resistant clones were then molecularly characterized.

The overall strategy for the screen is illustrated in Figure 1. The principle behind the screen is selection against retrovirally infected ES cells. The retrovirus used in the screen carried the *puro-Δtk *positive/negative selection marker [11]. Infected cells expressing the *puro-Δtk *fusion gene are sensitive to 1-(-2-deoxy-2-fluoro-1-β-D-arabino-furanosyl)-5-iodouracil (FIAU) negative selection; thus, mutant ES cells that cannot be infected by the virus will survive this negative selection. This screen is therefore strongly dependent on the ability to infect all cells in the culture with a retrovirus that reliably expresses a negative selection cassette. A very high infection efficiency must be achieved so that every single infectable cell in the culture has at least one infection event. In practice, the need to infect every cell in a culture of 10^9 ^cells requires superinfection, in which every cell has between 10 and 20 independent viral insertions. Superinfection can be achieved in a variety of ways, but in the screen described here it was accomplished by co-cultivation of the gene-trap ES cell library with the viral producer cells.

### Superinfection with a *puro-Δtk *retroviral vector

**Figure 2:**
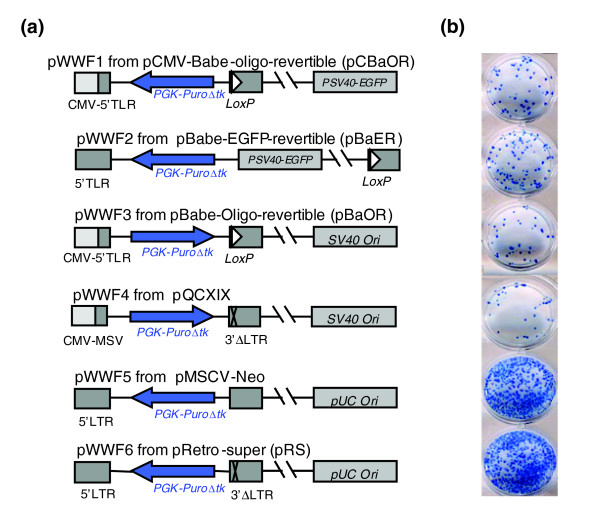
*Puro-Δtk *retroviral vector constructs and titres. **(a) **All vectors were constructed from murine leukemia virus (MuLV) backbones and contained the *Puro-Δtk *cassette. pWWF1, pWWF2, and pWWF3 are three revertible pBabe-based vectors containing a *loxP *site in the 3' long terminal repeat (LTR) [26]. pWWF4 and pWWF5 were constructed from vectors pQCXIX and pMSCV-Neo. pWWF6 was constructed in the pRetro-Super backbone. In some of these vectors the 3' LTR has a self-inactivating mutation provided by an internal deletion to generate a self-inactivating provirus. **(b) **Titers of transiently produced virus on ES cells after a single round of infection with 0.1 ml viral supernatant are illustrated by staining puromycin-resistant colonies in 24-well plates.

To generate a viral producer cell line with a high titre, six different murine leukemia virus (MuLV) backbones were tested. The *puro-Δtk *cassette was cloned into each backbone and the vectors were tested for their efficiency in producing recombinant virus by transient transfection into phoenix packaging cells [12]. Viral titers were assessed using wild type ES cells, and the WWF6 (Wang Wei female 6) vector had the highest titer (Figure 2) because of several point mutations and a deletion in its long terminal repeat that prevents transcriptional suppression in ES cells [13]. This recombinant vector was used to generate the stable viral producer cell line B4-5 in GPE-86 cells, another safe helper-free ecotropic packaging cell line [14], which produced a titre of 2 × 10^4 ^colony-forming units/ml.

**Figure 3:**
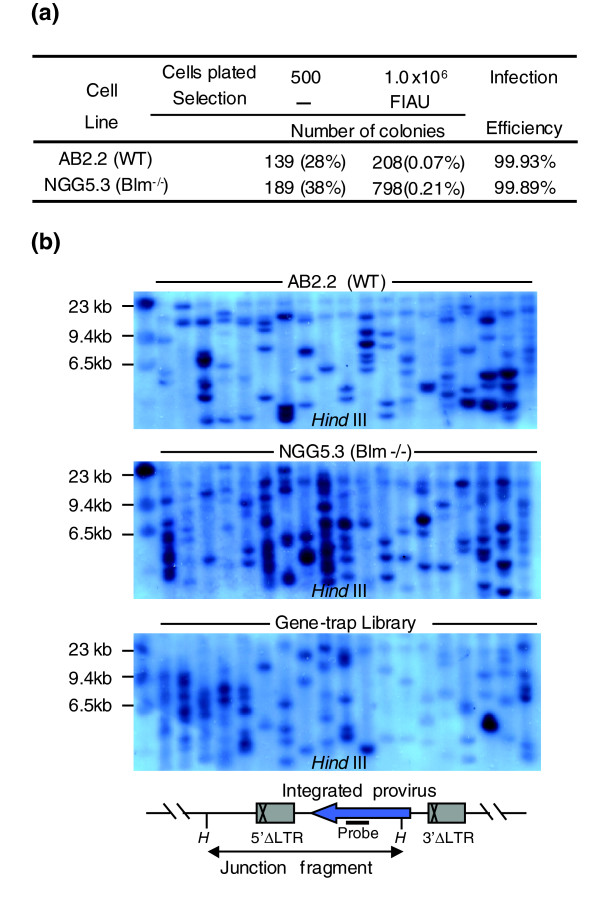
Super-infection of ES cells by co-cultivation. **(a) **Efficiency of infection by co-cultivation with the viral producer cell line B4-5. AB2.2, wild-type (WT), and NGG5.3 *Blm*-deficient embryonic stem cells cells were co-cultivated with irradiated B4-5 cells and plated in normal medium to determine plating efficiency in the absence of selection and 1-(-2-deoxy-2-fluoro-1-β-D-arabino-furanosyl)-5-iodouracil (FIAU) to measure the frequency of noninfected cells. **(b) **Assessment of proviral copy number in randomly picked nonselected wild-type and *Blm*-deficient embryonic stem (ES) cells after co-cultivation with the viral producer cell line B4-5. Southern blot analysis was performed by using *Hind*III digested genomic DNA isolated from different single cell clones cultured in nonselective medium. Each observed fragment represents a different proviral insertion. The probe is the *Pst*I fragment generated from *Puro-Δk *cassette. kb, kilobases; LTR, long terminal repeat.

Infection efficiency was investigated using both wild-type (AB2.2) and *Blm*-deficient (NGG5.3) ES cells using irradiated B4-5 producer cells. Following co-cultivation, 99.9% of the both NGG5.3 and AB2.2 ES cells were resistant to puromycin (Figure 3a). Furthermore, proviral copy numbers were investigated using clones isolated from these cultures without selection. Southern blot analysis was performed using an enzyme that cuts once in the provirus, and so each insertion site will have a unique proviral-host genome junction fragment (Figure 3b). This analysis revealed that, irrespective of genotype, all clones had multiple insertion events, with an average of five and a range from two to eight in this analysis. These comparisons confirmed that co-cultivation resulted in very efficient superinfection of *Blm*-deficient ES cells.

### Screening for infection resistant mutants

**Figure 4:**
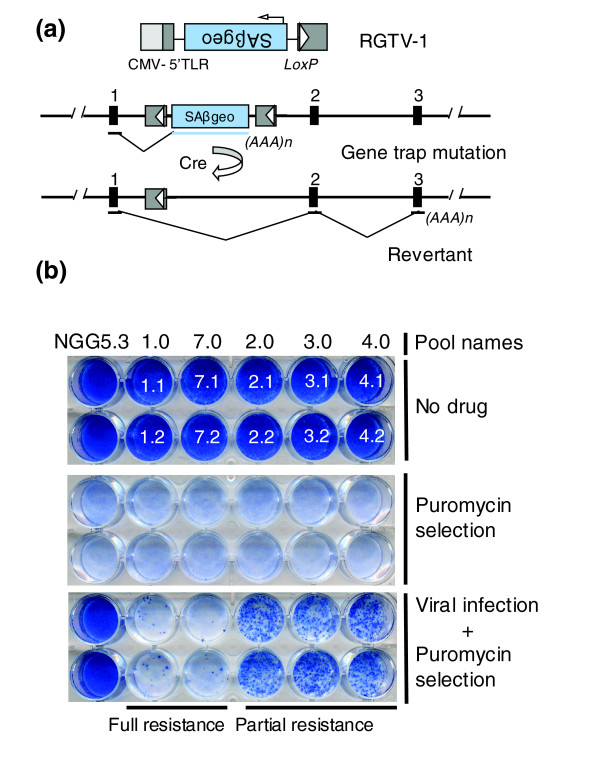
Retroviral infection resistant mutants. **(a) **Retroviral gene-trap mutagenesis. Illustration of the retroviral producer construct, RGTV-1, with the SAβgeo gene trap cassette in the retroviral vector backbone, an integrated provirus in the intron of a gene, and a Cre-reverted allele with a single long terminal repeat (LTR) retained in the locus. βgeo, lacZ-neo fusion gene; SA, splice acceptor. **(b) **Retroviral infection resistant phenotype of five independent mutant clones. Daughter embryonic stem (ES) cell clones from five different pools and controls were plated in triplicate in 24-well plates. 'No drug' indicates that all clones grew without selection; 'Puromycin selection' indicates that all clones were puromycin sensitive; and 'Viral infection + Puromycin selection' indicates that all clones were exposed to 1.2 × 10^5 ^colony-forming units of the B4-5 *puro-Δtk *retroviral vector followed by selection. The NGG5.3 controls were readily infected and clones from pools 1 and 7 were highly resistant to infection; clones from pools 2, 3, and 4 were less resistant to infection.

The gene-trap library used in this study is sectored into eight pools, each containing approximately 1,200 independent gene-trap clones recovered by G418 selection (Figure 4a). Each clone in the library has been expanded through a minimum of 14 doublings [9]. Given the total cell number, the number of cell divisions and the rate of mitotic recombination in *Blm*-deficient cells [8], each pool should contain a small number of homozygous mutant cells derived from each independent heterozygous insertion event.

The eight pools were separately co-cultivated with viral producer cells and then selected in FIAU. After the first round of FIAU selection, the surviving FIAU-resistant clones were challenged a second time with the *puro-Δtk *retrovirus to identify noninfectable mutant ES cells from those that were not infected by chance. After three rounds of selection, 178 infection-resistant clones were recovered from five of the eight pools. At the third round of selection, *Blm*-deficient ES cells (NGG 5.3) were included as a positive control because they are fully susceptible to viral infection. The phenotype of the infection-resistant clones was clearly different from that of the NGG5.3 cells (data not shown). Two representative clones from each of the five pools were randomly selected to test their degree of resistance to infection (Figure 4b). Among the mutant clones from the same pool the resistance level was quite similar, although it was variable between clones isolated from different pools. Clones from pools 1 and 7 were almost fully resistant to viral infection, whereas mutant clones from pools 2, 3, and 4 were partially resistant, although still obviously different from wild-type cells.

**Figure 5:**
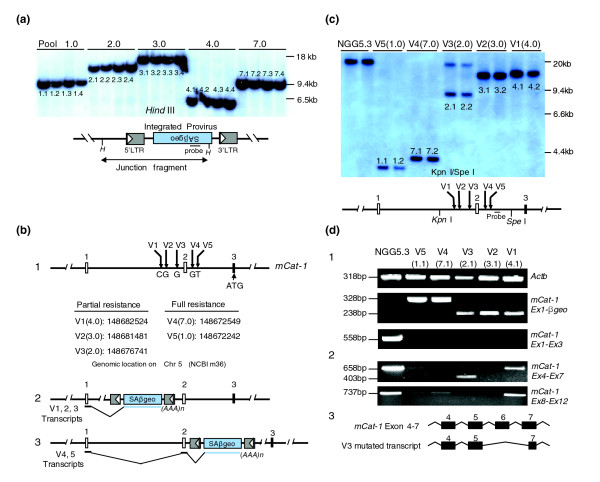
Molecular analysis of *mCat-1 *mutations. **(a) **Junction fragment analysis of retroviral resistant clones. Southern blot of retroviral infection resistant clones isolated from different subpools of the gene trap library. The clones isolated from different subpools have different *Hind*III proviral-host junction fragments and are thus independent mutants, as expected. The clones isolated form the same pools share a common host-proviral junction fragment and thus appear to be daughter clones in all cases. The probe is the *Cla*I fragment from the SA-βgeo cassette. LTR, long terminal repeat. **(b) **Insertion sites of gene-trap virus in *mCat-1*. Part 1 shows the structure of the 5' end of *mCat-1*; the initiating ATG codon is in exon 3. The proviral-host junction at the end of 5' LTR was sequenced by Splinkerette PCR. The location of the retroviral insertions is shown by arrows. Nucleotide positions are from National Center for Biotechnology Information (NCBI) mouse build 35. The pools that correspond to V1 to V5 are shown in brackets. The structure of the fusion transcripts for the insertions in introns 1 and 2 are also shown in parts 2 and 3, respectively. **(c) **Homozygosity analysis of *mCat-1 *in retroviral resistant clones. Southern blot with an *mCat-1 *probe. Absence of the wild-type fragment in V1, V2, V4, and V5 indicates that the viral insertions are homozygous. The presence of the wild-type fragment in the V3 clone reveals that in this clone the insertion is heterozygous. **(d) **Expression of wild-type and fusion transcripts in gene-trap mutants. As shown in part 1, reverse transcription (RT)-polymerase chain reaction (PCR) with primers for *mCat-1 *exon 1 and lacZ detected fusion transcripts of the expected size. Fusion transcripts of 238 base pairs (bp) were amplified from clones V1, V2 and V3, whereas transcripts of 328 bp were detected from clones V4 and V5. The wild-type exon 1 to 3 transcript was only amplified from *Blm*-deficient ES cells. β-Actin (*Actb*) served as a positive control for RT-PCR. As shown in part 2, RT-PCR with primers for *mCat-1 *exon 4 to 7 and exon 8 to 12 detected trace transcript levels of *mCat-1 *from mutant clones except V1. The 403 bp product detected by RT-PCR for exon 4 to 7 primers in the V3 clone was sequenced and found to have an aberrant splice, as illustrated in part 3.

To investigate the relationship between clones in same pools, 23 infection-resistant clones from pool 1 and 27 from pool 7 were analyzed by Southern blotting to detect the proviral-host junction fragment of the gene-trap vector using a SAβgeo probe. This analysis identified the same junction fragment in clones from the same pool (data not shown), implying that clones from the same pool were daughter clones. To determine whether this was the case for clones from all pools, a representative sample of four clones from each pool was tested (Figure 5a). In all cases, clones from the same pool exhibited the same junction fragment, verifying the above assumption. However, clones from the different pools had different junction fragments, confirming that they had independent mutations. A total of five independent clones were recovered, corresponding to five pools of the library.

### Cloning the viral insertion sites

The proviral-host junction from the five mutant clones was recovered using a splinkerette polymerase chain reaction (PCR) [15] and sequenced. The five sequences mapped to the *mCat-1 *gene, a known receptor of MuLV in fibroblasts [16]. The proviral-host junction sequences mapped to five different positions in introns 1 and 2 of the *mCat-1 *gene, demonstrating the independent origin of each of these five clones. The mutant clones from pools 1, 7, 2, 3 and 4 were termed V5, V4, V3, V2 and V1, respectively (Figure 5b). In all cases the SAβ geo cassette was in the appropriate transcriptional orientation. Because the ATG initiation codon of *mCat-1 *is in exon 3, translation of the βgeo fusion gene uses its own start codon. Furthermore, the integration sites of fully resistant clones V5 and V4 from pools 1 and 7 were downstream of exon 2 and closer to the ATG start condon, whereas the partially resistant clones were upstream of exon 2.

To determine whether the mutations were homozygous, these clones were purified by single cell subcloning and Southern analysis was performed using a probe from the *mCat-1 *gene. This identified different proviral/*mCat-1 *junction fragments in each clone, as expected (Figure 5c). Moreover, four of the mutant clones (V1, V2, V4 and V5) lacked the wild-type allele and were homozygous for the mutant allele, whereas the fifth mutant clone (V3) appeared to be heterozygous because it retained its wild-type *mCat-1 *allele.

To confirm the effect of the proviral insertions on expression of the *mCat-1 *locus, reverse transcription (RT)-PCR was performed using primers for exons 1 and 3, which spanned the proviral insertion sites (Figure 5d). In all cases (including the V3 clone), no product was detected. This confirmed that the viral insertions affected the generation of a wild-type *mCat-1 *transcript upstream of the normal start codon of *mCat-1*. Furthermore, the lack of a 'wild-type' exon 1 to 3 RT-PCR product in the V3 clone suggested that this clone may carry a mutation on the 'wild-type' allele that was not identified by Southern analysis. RT-PCR was performed to detect possible transcripts 3' of the proviral insertions. Using exon 4 to 7 and 8 to 12 primer pairs, an aberrant sized fragment was identified in the exon 4 to 7 RT-PCR product from clone V3 (Figure 5d), suggesting a potential splicing mutation on the 'wild-type' allele from this clone. Sequence analysis of this product revealed that the transcript of exon 4 to 7 from clone V3 was shortened by skipping of exon 6 and part of exon 7 (Figure 5d), providing an explanation for the viral resistance of this clone.

### Confirmation of the causality of the mutations by Cre reversion

**Figure 6:**
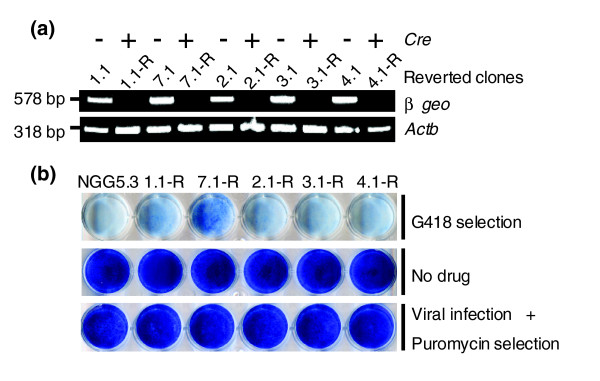
Reversibility of *mCat-1 *mutations with Cre. **(a) **Genomic polymerase chain reaction (PCR) with primers for *LacZ *and *Neo *detected the βgeo cassette in the five *mCat-1 *mutations but was absent in five revertants. β-Actin (*Actb*) served as a positive control for genomic PCR. **(b) **Five revertant clones were sensitive to G418, confirming excision of the virus and susceptibility to retroviral infection.

Southern blotting analysis using a SAβgeo probe confirmed that each of the five gene-trap clones had only a single gene-trap viral insertion (Figure 5a), although in four out of five cases this was bi-alleleic. To verify that the gene-trap insertions in *mCat-1 *were directly responsible for the observed phenotype, these were reverted with Cre, which deletes the provirus, leaving a single long terminal repeat in the locus. Reverted clones (1.1R, 7.1R, 2.1R, 3.1R, and 4.1R) were identified following transient expression of Cre by G418-sib selection and confirmed by genomic PCR, with primers for the βgeo cassette (Figure 6a). Excision of the proviral insertion restored the retroviral infection sensitivity of the revertants to wild-type levels for each of the five clones tested (Figure 6b).

## Discussion

Successful infection by a retrovirus requires many host cell factors [2]. One of the most important of these is the receptor present on the cell surface, which is necessary for retroviral entry into the cell. Receptor-retroviral interactions can be quite complex and may involve more than one molecule; for instance, HIV requires a co-receptor in addition to CD4 [4]. *mCat-1*, which encodes a cationic amino acid transporter, was identified as the receptor for murine ecotropic leukemia viruses by an expression cloning strategy in fibroblasts [17]. In our study we used a loss-of-function assay, based on the principle of negative selection of infected cells leading to the recovery of resistant cell clones. Because we are using a retroviral vector to screen for mutants, the event that the *mCat-1 *mutation blocks should occur at an early stage of the retroviral life cycle, between receptor binding and integration into the genome. Given previous data from other cell lines, we believe that our identification of *mCat-1 *as the major MuLV receptor in ES cells is reasonable.

In our screen, we recovered five independent mutations of *mCat-1 *in a library of 10,000 independent gene-trap mutations. The fact that we did not identify any other critical gene in this screen illustrates that receptor-mediated entry is perhaps the most nonredundant, essential step in the retroviral replication. Previously, Murray [18] and Sheng [19] and their coworkers exploited gene-trap mutagenesis to generate mutation libraries in several mammalian cell lines, and using these libraries they identified genes required for viral replication of HIV-1, filoviruses, and reovirus These studies confirm that insertional mutagenesis provides a rapid, genome-wide approach to identifying host cellular factors that are required for virus infection in different cell types.

The recovery of multiple independent mutations in the *mCat-1 *gene in this study confirms its importance in MuLV replication. This library was used previously to screen for genes in the DNA mismatch repair pathway [9]. In the previous screen seven independent homozygous mutations in *Msh6 *and two independent mutations in *Dnmt1 *were recovered. The number of independent mutations recovered in the *Msh6 *and *mCat-1 *genes is greater than expected based on the complexity of the library, which suggests that these genes might be integration 'hot spots' for the gene-trap vector used as the insertional mutagen for this library. A systematic analysis of gene-trap 'hot spots' has been described by the German Gene Trap Consortium (GGTC), which reported that 75% of gene-trap mutations appeared only once in the gene-trap database but 25% were represented by multiple clones and half of these 'hot spots' were vector specific [20]. Thus, our previous failure to identify mutations in *Msh2 *and *Mlh1 *mismatch repair MMR genes in our previous study, as well as other retroviral-related host factor genes in this study, suggests that the use of a single retroviral vector limited the coverage of the genome.

More than 10,000 genes are known to be expressed and mutable by gene trapping in ES cells [21]. Promoter gene-trap mutagenesis is dependent on correct splicing between the endogenous gene and the βgeo splice acceptor, and either the generation of a fully functional fusion protein with the trapped gene or insertion upstream of the normal initiation codon. In this study only one potential reading frame of the gene-trap virus was used, which limits the selectable insertion events to those that occur in the appropriate reading frame and produce a functional fusion protein. The gene-trap fusion transcripts with *mCat-1 *and those isolated previously (*Msh6 *and *Dnmt1*) were in the same reading frame. In principle, we should be able to recover additional genes required for retroviral infection by constructing and screening libraries with gene-trap vectors with alternative reading frames. Another limitation of retroviral mutagenesis is the nonrandom nature of retroviral integration, which is known to favor the 5' ends of genes. Vectors such as Piggybac [22] provide alternative gene-trap insertional mutagenesis methods that potentially are without such a bias. Finally, the chance of generating a homozygous mutation in *Blm*-deficient ES cells will depend on its chromosomal position. Homozygous mutations are more likely to be generated in genes located closer to the telomere than those close to the centromere. Indeed, *mCat-1 *is located at 148.7 megabases (Mb), just 4 Mb from the telomere of chromosome 5, whereas the gene recovered most frequently in our previous screen [8], namely *Msh6*, is located at 88.7 Mb, just 7 Mb from the telomere of chromosome 17. In contrast, *Dnmt1 *is located at 20.7 Mb, suggesting that the reduced frequency of recovering mutations in *Dnmt-1 *in previous studies may be related in part to its chromosomal location.

In order to avoid some of the biases associated with gene-trap mutagenesis, highly efficient mutagenesis agents such as γ-irradiation or chemical mutagens can be used. Indeed, ENU (*N*-ethyl-*N*-nitrosourea) was successfully used to conduct a screen with *Blm*-deficient ES cells previously [10]. However, with such approaches identification of the molecular change can be extremely difficult, and in this respect retroviral vector or transposon based gene-trap approaches offer a major advantage. cDNA expression libraries provide an alternative for functional screening for host genes that confer susceptibility to viral infection. Such screens are generally configured to rescue viral resistance of a cell line, which might operate at any stage of the replicative cycle of the virus [23,24].

## Conclusion

In a summary, in this screen we exploited an approach that combined gene-trap insertional mutagenesis in *Blm*-deficient ES cells with superinfection and negative selection, and proved that *mCat-1 *is an essential host factor for retroviral infection in ES cells. In principle, application of this screening methodology with more complete libraries should identify other cellular factors that are required in the early stages of retroviral infection. Moreover, although the coverage of the existing library is not complete, it should prove valuable for recovery of essential host factors for other pathogenic agents.

## Materials and methods

### Construction of retroviral vectors

To generate a retroviral vector with the highest possible titre, the *puro-Δtk *positive/negative selection cassette from pYTC37 [11] was cloned into several different retroviral vector backbones. The backbones used were pCMV-Babe-Oligo-Revertible (pCBaOR), pBabe-EGFP-Revertible (pBaER), and pBabe-Oligo-Revertible (pBaOR); these three vectors were modified on pBabe retroviral vectors [25,26], pQCXIX and pMSCV-Neo (retroviral expression vectors; Clontech, Mountain View, CA, USA) [27,28], and pRetro-Super (pRS; a gift from Roderick Beijersbergen, The Netherlands Cancer Institute, Amsterdam, The Netherlands.)

### Embryonic stem cell culture

ES cell culture was described in detail previously [30]. Briefly, ES cells were maintained on γ-irradiated feeder cell layers in Dulbecco's modified Eagle's medium supplemented with 15% fetal bovine serum, 2 mmol/l L-glutamine, 50 units/ml penicillin, 40 μg/ml streptomycin, and 100 μmol/l β-mercaptoethanol. Cells were cultured at 37°C with 5% carbon dioxide.

### Transient titer test

The titer of each retroviral vector was assessed by transient transfection of 25 μg of each vector into phoenix helper-free packaging cells [12] using calcium phosphate transfection [31]. Packaged virus was harvested 48 hours after transfection.

The viral titer was assessed using ES cells. Twenty four hours before infection, ES cells were plated in 24-well plates at a density of 3 × 10^5 ^cells per well. Viral supernatant from each vector was filtered through a 0.45 μm filter, and polybrene (hexadimethrine bromide) was added to a final concentration of 10 μg/ml. One milliliter of each filtered supernatant was applied to ES cells, and puromycin selection (3 μg/ml) was initiated 24 hours after infection and continued for 8 days. Drug-resistant ES colonies were fixed and stained with 2% methylene blue in 70% ethanol and counted.

### Construction of a stable viral producer cell line

pWWF6 (25 μg) was transfected into 1 × 10^7 ^GPE-86 cells [14] by electroporation (290 V/cm and 960 μF). The cells were plated and puromycin was added 48 hours after electroporation. Puromycin-resistant colonies were picked into 24-well plates and the titres of 72 independent colonies were assessed as described previously. The 10 clones with the highest titers were reassessed to identify one for use in future experiments. The clone with highest titer was B4-5.

### Superinfection of gene-trap library

The screen relies on superinfection of the subpools of the gene-trap library. To maximize exposure of the ES cells to viral particles, a co-cultivation strategy was used. B4-5 cells were collected, suspended in media at a density of 1 × 10^7 ^cells/ml, and γ-irradiated with the dose of 6000 cGray. About 6 × 10^7 ^irradiated B4-5 cells were plated on to a 150 mm tissue culture dish and 24 hours later 3.5 × 10^6 ^ES cells from each pool of the gene-trap library were plated onto the irradiated B4-5 cells. After six days of co-culture, the ES cells were confluent. These cells were expanded and 1.8 × 10^7 ^cells were selected in FIAU (0.2 μmol/l). Selection was maintained for 10 days. FIAU-resistant ES cell colonies were picked into 96-well feeder plates for further assessment.

### Second and third round infection assay

Second and third round infection was performed respectively in 96-well and 24-well feeder plates using a sib-selection strategy. For the second round of infection, each clone was plated in duplicate 24 hours before infection and one duplicate was exposed to 200 μl (approximately 4 × 10^3 ^colony-forming units) of viral supernatant from B4-5 cells. This was repeated 12 hours later. The following day one plate was selected with puromycin and a duplicate copy was maintained without selection. Clones that were infected were resistant to puromycin and excluded. Puromycin-sensitive clones were then tested a third time in 24-well plates using the same protocol to confirm their resistance to infection.

### Isolation of the proviral junction

Proviral junction fragments were isolated using Splinkerette-PCR, as described elsewhere [15]. Briefly, genomic DNA was digested with *Sau3A*I or *Fat*I and ligated with the corresponding Splikerette adaptors HMSp-*Sau3A*I or HMSp-*Fat*I. The Splikerette adaptors were generated by annealing Splikerette oligoes HMSpBb-*Sau3A*I or HMSpBb-*Fat*I with HMSpAa. The first found of PCR was carried out with viral primer AB949new and Splinkerette primer HMSp1. The nested PCR was carried out with the viral primer HM001 and Splinkerette primer HMSp2. The specific PCR products were gel purified and TA cloned [32] for sequencing.

### Southern blotting and hybridization

Total genomic DNA was restricted, size fractionated on agarose gels, blotted, and hybridized using standard procedures. The following probes were used: *LacZ*, a 800 base pair *Cla*I-digested fragment from pSAgeo [33], which is a plasmid containing the *SAβgeo *cassette in pBS; and *Puro-Δtk*, a 1.2 kilobase *Pst*I-digested fragment from pYTC37 [11], which is the plasmid that contains the *puro-Δtk *cassette in pPGKbpA.

### Reverse transcription polymerase chain reaction

Total RNA was extracted from the five mutant clones and NGG5.3 cells as a negative control, and 5 μg total RNA was used to generate the first strand cDNA. β-Actin (*Actb*) was used as a positive control for RT-PCR. The PCR product of the mutant *mCat-1 *transcript from exon 4 to 7 from clone V3 was TA cloned for sequencing.

### Cre reversal

Five million ES cells were electroporated with 10 μg of the Cre expression plasmid pCAG-Cre [34], plated at low density (2,000 cells/90 mm plate), and grown without selection for 9 days. ES cell colonies were picked into 96-well plates. Clones that had excised both copies of the gene-trap cassette were identified as G418-sensitive clones by sib-selection and confirmed by genomic PCR, using primers that amplify a 578 base pair fragment in the *βgeo *cassette. Reinfection was performed as described above to assess phenotypic reversal.

## Figures and Tables

**Table 1:** Sequence of nucleotides and primers.

Nucleotide/primer	Sequence
Splinkerette nucleotides	
HMSpAa	5'-CGA AGA GTA ACC GTT GCT AGG AGA GAC CGT GGC TGA ATG AGA CTG GTG TCG ACA CTA GTG G-3'
HMSpBb-*Sau3A*I	5'-gat cCC ACT AGT GTC GAC ACC AGT CTC TAA (T)10C(A)7-3'
HMSpBb-*Fat*I	5'-cat gCC ACT AGT GTC GAC ACC AGT CTC TAA (T)10C(A)7-3'
Splinkerette PCR primers	
AB949new	5'-GCT AGC TTG CCA AAC CTA CAG GTG G-3'
HM001	5'-GCC AAA CCT ACA GGT GGG GTC TTT-3'
HMSp1	5'-CGA AGA GTA ACC GTT GCT AGG AGA GAC C-3'
HMSp2	5'-GTG GCT GAA TGA GAC TGG TGT CGA C-3'

Primers for sequencing	
M13 forward primer	5'-CTG GCC GTC GTT TTA C-3'
M13 reverse primer	5'-CAG GAA ACA GCT ATG AC-3'

RT-PCR primers	
Oligo-dT primer	5'-GGC CAC GCG TCG ACT AGT AC (T)_17_-3'
mCAT1-exon1-F	5'-GCG TGC GCC ATC CCC TCA GCT AGC A-3'
mCAT1-exon3-R	5'-CGA TGA TGT AGG AGA GAA TCA GGT-3'
mCAT1-exon4-F	5'-GTA CTT CAA GCG TGG CAA GAG-3'
mCAT1-exon7-R	5'-CTG GTG GAA AGT GCG CAG AGA-3'
mCAT1-exon8-F	5'-TCT CCT AGG CTC CAT GTT TCC C-3'
mCAT1-exon12-R	5'-CTA TCA GCA TCC ACA CTG CAA A-3'
LacZ-Gsp2-R	5'-ATG TGC TGC AAG GCG ATT AAG-3'

Genomic PCR primers	
Actb-exon4-up	5'-GTT TGA GAC CTT CAA CAC CCC-3'
Actb-exon4-down	5'-GTG GCC ATC TCC TGC TCG AAG TC-3'
zk274_in b-geo	5'-CGCCCCTGCGCTGACAGCCGGAACACGGCG-3'
zk276RC_in b-geo	5'-AACTGCCAGCTGGCGCAGGTAGCAGAGCGG-3'

PCR, polymerase chain reaction; RT, reverse transcription.
